# Rethinking childhood adversity in chronic fatigue syndrome

**DOI:** 10.1080/21641846.2018.1384095

**Published:** 2017-10-10

**Authors:** James E. Clark, Sean L. Davidson, Laura Maclachlan, Julia L. Newton, Stuart Watson

**Affiliations:** ^a^ Wolfson Research Unit, Newcastle University, Newcastle, UK; ^b^ Newcastle Hospitals, NHS Foundation Trust and Newcastle University, Institute for Cellular Medicine, Newcastle, UK; ^c^ NTW NHS Foundation Trust, Newcastle, UK

**Keywords:** Childhood adversity, chronic fatigue syndrome, modelling, childhood trauma, depression

## Abstract

**Background:** Previous studies have consistently shown increased rates of childhood adversity in chronic fatigue syndrome (CFS). However, such aetiopathogenic studies of CFS are potentially confounded by co-morbidity and misdiagnosis particularly with depression.

**Purpose:** We examined the relationship between rates of childhood adversity using two complimentary approaches (1) a sample of CFS patients who had no lifetime history of depression and (2) a modelling approach.

**Methods:** Childhood trauma questionnaire (CTQ) administered to a sample of 52 participants with chronic fatigue syndrome and 19 controls who did not meet criteria for a psychiatric disorder (confirmed using the Structured Clinical Interview for DSM-IV). Subsequently, Mediation Analysis (Baye’s Rules) was used to establish the risk childhood adversity poses for CFS with and without depression.

**Results:** In a cohort of CFS patients with depression comprehensively excluded, CTQ scores were markedly lower than in all previous studies and, in contrast to these previous studies, not increased compared with healthy controls. Post-hoc analysis showed that CTQ scores correlated with the number of depressive symptoms during the lifetime worst period of low mood. The probability of developing CFS given a history of childhood trauma is 4%, a two-fold increased risk compared to the general population. However, much of this risk is mediated by the concomitant development of major depression.

**Conclusions:** The data suggests that previous studies showing a relationship between childhood adversity and CFS may be attributable to the confounding effects of co-morbid or misdiagnosed depressive disorder.

**Abbreviations:** CFS: Chronic fatigue syndrome; CTQ: Childhood trauma questionnaire; MDD: Major depressive disorder; CA: Childhood adversity; *P*: Probability

## Introduction

Childhood adversity refers to a range of experiences which are detrimental to normal development. This includes explicit episodes of abuse as well as more chronic neglect, and can be physical or emotional. It is a key regulator in the development of neuroendocrine and immune systems and is implicated in the vulnerability to, and maintenance of, physical and psychiatric disorders. A number of existing case–control studies have used retrospective questionnaires to assess the prevalence of childhood trauma in chronic fatigue syndrome (CFS) [1]. With the exception of one small study by Taylor and colleagues [2], all have demonstrated a significantly increased prevalence of one or more subtypes of childhood trauma in people with CFS which has led some to hypothesise that early childhood adversity is a risk factor for CFS [3–5]. Previous work has, however, neglected the potential mediating impact of depressive symptoms on this finding. Our hypothesis is therefore that the increased prevalence of childhood adversity (CA) is related to the high comorbidity with major depressive disorder found in this patient group rather than being a primary risk factor for CFS itself.

In order to explore the hypothesis that childhood adversity increases the risk of CFS predominantly via its effects on the risk of depression, we determined the prevalence of early adversity in a CFS population who were free of current or lifetime depression and applied this and existing data to a novel mediation analysis using Bayes’ rule which allows us to establish the risk that childhood adversity poses for the development of CFS both with and without concomitant depression.

## Methods

The study was performed in two phases. Phase 1 was a cross sectional case–control study and phase 2 was a modelling study performed using mediation analysis. The study was carried out in accordance with the latest version of the Declaration of Helsinki. The study design was approved by the Newcastle and North Tyneside Ethics Committee and all participants provided written informed consent.

### Phase 1: cohort study

#### Participants

52 participants fulfilling the Fukuda criteria for CFS [6] and 19 healthy controls were recruited between as part of an MRC Funded cohort study (MRC MR/J002712/1). Participants with CFS were recruited from the UK Department of Health Newcastle and North Tyneside CFS Clinical Service and controls were recruited predominantly through a volunteer database. Inclusion criteria consisted of a diagnosis of CFS and age 18 years or over. Exclusion criteria were current or life time diagnosis of major depressive disorder.

#### Measures

All participants underwent psychiatric diagnostic assessment using the Structured Clinical Interview for DSM-IV (SCID-I) [7] which was carried out in all cases by LM supported and trained by SW. As part of this, all participants were asked to consider their lifetime worst period of low mood or loss of interest. The number of depressive symptoms coded as *threshold* or *subthreshold* during this period was recorded; any participants meeting criteria for current or past major depressive disorder (MDD) or other axis I psychiatric disorder were excluded.

Childhood adversity was assessed using the Childhood Trauma Questionnaire (CTQ), a 28 item self-administered questionnaire which provides a retrospective measure of five subtypes of childhood experiences: emotional neglect; emotional abuse; physical neglect; physical abuse; and sexual abuse. Each subtype of abuse is assessed by five items; three additional items assess tendency of respondents to minimise or deny abuse experiences. Participants rate the truth of each statement on a one to five scale from ‘Never’ to ‘Very often’ true when they were growing up. Thus for each subtype, the minimum score is five and the maximum is 25. Cut-off scores based on Bernstein and Fink’s CTQ manual were used to group scores into low to moderate, moderate to severe and severe to extreme severity [8]. The CTQ has previously been shown to fit a 5 factor model and to have acceptable internal consistency [9].

#### Ethical permission

The study was approved by the NRES Committee North East – Newcastle & North Tyneside 2. All participants gave written informed consent.

#### Analysis

The CFS data was positively skewed and not amenable to transformation hence non-parametric statistics were used, specifically the Chi-squared and Mann-Whitney U tests for comparisons of categorical and continuous variables respectively. Spearman’s rho was used for correlational analysis.

### Phase 2: mediation analysis

Mediation analysis makes use of Bayes’ rule, which is a simple method for calculating the conditional probability of an event (*x*) given another event (*y*). This is termed the posterior and is given by:

P(x|y)

In words, the posterior is therefore ‘the probability that *x* occurs given *y* has occurred’; an equivalent way of saying this is ‘the probability that if *y* occurs then *x* occurs’. According to Bayes’ rule, the posterior is given by the following equation:

P(x|y)=P(x)⋅P(y|x)/P(y)

*P*(*y*|*x*) is the likelihood and is ‘the probability of *y* given *x*’. *P*(*y*) is the ‘the probability of *y*’ and is termed the model evidence. *P*(*x*) is ‘the probability of *x*’ and is the prior.

The fundamental tenet of Bayes’ rule is its treatment of probability from an epistemic standpoint, where the prior represents an initial belief in the outcome of an event before exposure to some new information. This new information is given by the likelihood over the evidence and can be thought of as the extent to which *y* is uniquely associated with *x*, so if *y* is likely to occur without *x*, or if *y* is less likely to occur after *x* has happened the posterior will be less than the prior. Conversely, if the presence of *x* increases the probability of *y* the posterior will be greater. The posterior is therefore an updated belief in a given outcome after exposure to novel information.

Bayes’ rule is therefore a powerful tool for reasoning about the probabilities of given outcomes in particular demographic groups. As such, it allows us to calculate the three probabilities mentioned above in turn, thereby constructing a novel mediation analysis to explore the risk that childhood adversity poses for the development of CFS, both with and without concomitant MDD.

In order to construct our mediation analysis we considered the following probabilities:

- That an individual currently has, or has had, a diagnosis of MDD given they have a threshold or higher score on measures of CA
- That an individual is diagnosed with CFS given they have a lifetime history of MDD
- That an individual has a diagnosis of CFS given no lifetime history of MDD and a threshold or higher score on measures of CA

## Results

### Phase 1: cohort study

In the CFS group there were 39 females and 13 males and the mean age was 45.9 years (s.d. = 11.8). In the control group there were 12 females and 7 males and the mean age was 45.1 (s.d. = 16.9). Groups were matched for gender (*χ* ^2^ = 0.96, *df* = 1, *p *= 0.38) and age (U = 491.5, *p* = 0.974). CTQ total scores and scores for all sub-scales did not differ between participants in the CFS and control groups (Table 1).

**Table 1.** CTQ scores of participants with CFS compared with controls, correlation between CTQ and SCID-I domain scores and the prevalence of childhood trauma in people with CFS in this and previous studies.^a^

CTQ trauma subtype	Mean CTQ scores		Mann-Whitney Comparison	SCID-I number of subthreshold and threshold symptoms	SCID-I number of threshold symptoms only	Prevalence of childhood trauma by sub-scale^b^	
	CFS*N* = 52	Controls *N* = 19	*p*	*r* (*p*)	*r* (*p*)	This study	Previous studies [10–12]
Emotional abuse	7.0	6.8	0.59	0.32 (0.007)	0.32 (0.007)	5.8%	24.7–42.0%
Physical abuse	5.3	5.4	0.96	−0.06 (0.64)	−0.098 (0.32)	0.0%	24.7–33.0%
Sexual abuse	5.5	5.1	0.91	−0.08 (0.51)	−0.03 (0.78)	3.8%	28.0–33.0%
Emotional neglect	7.8	7.3	0.64	0.36 (0.003)	0.29 (0.01)	7.7%	25.5–60.0%
Physical neglect	5.7	5.3	0.96	0.31 (0.008)	0.24 (0.048)	3.8%	14.0–24.5%
Total CTQ score	31.2	29.8	0.80	0.38 (0.001)	0.32 (0.006)	17.3%^c^	

^a^Table showing the mean CTQ scores of CFS and control groups with a comparison using the Mann-Whitney U test; the Spearman’s rho correlational analysis between CTQ scores and number of depressive symptoms recorded as *subthreshold* or *threshold* and *threshold* only on the SCID-I in all participants; and the proportion of participants with CFS meeting the moderate to severe CTQ cut-off scores for each sub-scale in this and previous studies.

^b^In line with previous studies, participants were considered to have a particular type of childhood trauma if they met the moderate to severe CTQ cut-off score for that sub-scale; cut-offs are from Bernstein and Fink’s CTQ manual [8].

^c^The figure 17.3% is the percentage of CFS patients who meet moderate to severe or severe to extreme cut-offs for any sub-scale.

CTQ: Childhood Trauma Questionnaire.

SCID-I: Structured Clinical Interview for DSM-IV Research Version.

*U*: Mann Whitney U.

*r:* Spearman’s rho correlation coefficient.

*Post hoc* analysis involving all participants revealed a significant correlation between the numbers of depressive symptoms coded as either *threshold* or *subthreshold* on the SCID-I with CTQ total score and CTQ sub-scale scores for emotional abuse, emotional neglect and physical neglect. The analysis also revealed that the number of *threshold* symptoms significantly correlated with CTQ total score and CTQ scores for the emotional abuse and emotional neglect sub-scales (Table 1).

### Phase 2: mediation analysis

We calculated **probability 1** using Bayes’ rule as follows:

$$P(\text{MDD}|\text{CA})=\;\frac{P(\text{CA}|\text{MDD})}{P(\text{CA})}P(\text{MDD})$$

This is the probability that an individual has received a diagnosis of MDD at some point throughout their lifetime if they have suffered adversity during childhood. The prior is simply the lifetime prevalence of depression, for which 16% is a reasonable estimate [13]. The evidence in this case is the probability of having experienced CA, which a large study of 1007 representative healthy individuals has placed at 35% [14]. The likelihood is the probability of an individual with depression having suffered adversity in childhood, which generally varies widely across studies, though a recent study has placed this at 75% according to cut-off scores on the childhood trauma questionnaire (CTQ) [15]. We therefore calculate the posterior:

$$P(\text{MDD}|\text{CA})=\frac{0.75}{0.35}0.16=0.34$$

This means we would expect 35% of individuals who meet threshold criteria for childhood adversity to have a diagnosis of MDD- a roughly twofold increase on baseline prevalence.

**Probability 2** is given by:

$$P(\text{CFS}|\text{MDD})=\;\frac{P(\text{MDD}|\text{CFS})}{P(\text{MDD})}P(\text{CFS})$$

This is the probability of an individual having a lifetime diagnosis of CFS if they have received a diagnosis of MDD. The prior is the lifetime prevalence of CFS which is estimated at 2% [16]. The evidence, as mentioned above, is 16%. The likelihood in this case is the probability of having depression given a diagnosis of CFS which has been placed at 67% [17]. We therefore calculate:

$$P(\text{CFS}|\text{MDD})=\frac{0.67}{0.16}0.02=0.08$$

Current evidence, examined using Bayes’ Rule therefore shows that there is an 8% chance of an individual receiving a diagnosis of CFS if they have MDD.

**Probability 3** is the probability that an individual receives a diagnosis of CFS, given they have no history of MDD but have experienced CA. In formal notation this is given by:

P(CFS|(−MDD|CA))

According to [18] this is equal to the probability that the individual has CFS given no history of MDD and a history of adversity such that:

P(CFS|(−MDD|CA))=P(CFS|(−MDD,CA))

This simplifies calculation of probability 3 somewhat:

$$P(\text{CFS}|-\text{MDD},\text{CA})=\;\frac{P(-\text{MDD},\text{CA}|\text{CFS})}{P(-\text{MDD},\text{CA})}P(\text{CFS})$$

The evidence here, i.e. *P*(−MDD, CA) is the probability that someone in the general population will not have depression and will have life adversity). This can be calculated as the probability that someone who has experienced CA will not also have depression multiplied by the probability that someone in the general population will have ELA.

P(−MDD,CA)=P(−MDD|CA)P(CA)

We have already shown that 35% of individuals who have experienced CA will develop MDD and can therefore say that 65% will not develop MDD. Therefore *P*(−MDD/CA) = 0.65. We have already stated that *P*(ELA) is 0.35. Therefore:

P(−MDD,CA)=0.65⋅0.35=0.23

This means that the probability of experiencing CA and not having a diagnosis of MDD is 23%.

Returning to probability 3, we have already stated that the prior in this case (*P*(CFS)) is 2%.

Estimates for the likelihood P(−MDD,CA|CFS) are sparse, given most studies have not routinely screened for history of depression. For this value we use data from our study which showed, in a sample of patients with no history of depression, prevalence of ELA was 17% (see Table 1) across all trauma domains. Substituting these values into Bayes’ rule we obtain:

$$P(\text{CFS}|-\text{MDD},\text{CA})=\;\frac{P(-\text{MDD},\text{CA}|\text{CFS})}{P(-\text{MDD},\text{CA})}P(\text{CFS})$$

$$P(\text{CFS}|-\text{MDD},\text{CA})=\;\frac{0.17}{0.23}0.02$$

P(CFS|(−MDD|CA))=0.015

This shows that if an individual experiences CA, but does not develop MDD there is a 1.5% risk that they will develop CFS.

**Figure 1.**
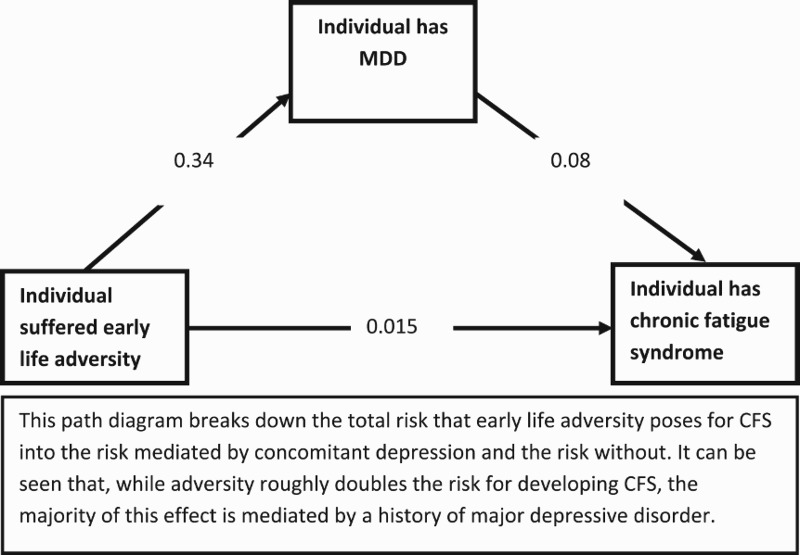
Path diagram.

Having calculated all three probabilities we can construct the following path diagram (Figure 1).

Considered as a whole this diagram shows that the total probability (through both paths) of having CFS given a history of CA is (0.34*0.08) + 0.015 = 4% – a twofold increased risk compared to the general population (Note that this is slightly lower than Heim et al. due to generally higher prevalence of CFS estimated in their study). However, the majority of this risk (3%) is mediated through a path including concomitant MDD. It is therefore of little surprise that conclusions regarding the aetiopathogenic role of CA vary according to the proportion of patients with MDD. Indeed, a quick calculation shows that prevalence of adversity in patients who have not been screened is expected to be very high (66%); interestingly this is almost exactly in line with two large and well-designed studies [6,7]. Our results show that by removing the increased risk mediated by depression this figure drops to 17%.

## Discussion

This case control study revealed that reported history of childhood trauma did not differ between people with CFS and matched controls, this finding, together with the Bayesian analysis support our hypothesis that childhood adversity increases the risk of CFS predominantly via its previously demonstrated effects on the risk of depression.

The inferential method used is a novel way of decomposing the risk conferred by CA into direct and mediated effects. The use of Bayes’ rule requires no assumptions about data, simple calculations and subsequent interpretation is relatively simple; the validity of the inferences is therefore robust. We acknowledge that soundness of the values used could be disputed, though all figures were either taken from published sources or represent widely accepted and quoted prevalence rates- indeed in some cases our estimates may be conservative. This simple but effective analysis demonstrates that a sizeable proportion of the risk posed by CA for developing CFS is not direct.

The elevated CTQ scores seen in previous CFS studies have been reported as confirmatory evidence that early adversity is an important risk factor for CFS, an interpretation which has become clinical lore and which influences perception of the aetiology of CFS and arguably increases stigmatisation. Data in this current study however suggests that a degree of caution is needed when considering this interpretation. Consideration needs to be given to the hypothesis that childhood adversity does not increase vulnerability to CFS and that the high rates of adversity seen in previous studies of CFS [1,2,10,11,19,20] are mediated by concomitant depression.

It is noteworthy that CFS shares many symptoms with depression and the two are frequently co-diagnosed [10,11,21]. Despite this, the current study appears to be the only examination of childhood trauma in CFS which rigorously excluded people with MDD and other psychiatric disorders. This may explain the contrast with existing literature; a view which is supported by the post hoc analysis in the case-control study which demonstrated that in all participants CTQ total score and CTQ scores for emotional abuse, emotional neglect and physical neglect subtypes correlated with the number of depressive symptoms during lifetime worst period of low mood or loss of interest in patients who did not fulfil criteria for depression (Table 1). This result is in keeping with two previous population based studies of people with CFS which reported a positive correlation between measures of childhood adversity and measures of current depressive symptoms [10,11].

Future studies investigating the impact of childhood adversity in CFS should acknowledge the substantial effect of MDD and depressive symptoms on this relationship, either by careful screening of participants or through simultaneous rating of depression. We suggest that, given the important role of depression in CFS, a re-evaluation of the hypothesised aetiological role of adversity is required. This entails formal investigation preferably involving direct comparison of patients with and without comorbid MDD.

Alternatively, the negative result in our case-control study may be a type II error, the risk of this is increased by the relatively small sample size particularly the small number of healthy controls (*n* = 19). It is noteworthy however that the rates of adversity in this current study were in keeping with rates of adversity in comparators recruited in our centre for other clinical studies [22]. Our *post hoc* analysis must also be interpreted with caution because the clustering of CTQ and SCID-I results around low scores could potentially have created a false correlation. The SCID-I is a diagnostic tool; if participant’s responses are not coded as *threshold* or *subthreshold* when asked about low mood or loss of interest, they are not asked about the other seven depressive symptoms on the SCID-I. Thus not all of our participants were asked about every symptom. The confounding effect of MDD on studies of childhood trauma may be mediated both by the aetiological role that early adversity plays in the pathogenesis of depression [12,23] and also by the potential for MDD to engender negative recall bias of past trauma and hence inflate CTQ scores [24,25].

Further analysis of existing CFS studies which have recorded, but not excluded, co-morbid MDD [10,11], ideally using meta-analytic tools to utilise data from the available trials, would be of interest, however, a larger study which compares reported childhood adversity between CFS patients with and without MDD and with a healthy comparator group may be required in order to address the question of whether, as has been previously assumed, childhood adversity is a significant risk factor for CFS. This question affects our understanding of the aetiology of CFS and also has implications for the interpretation of pathophysiological studies in CFS which may be equally confounded by inclusion of participants with co-morbid MDD.
